# Mechanistic and Kinetic Differences between Reverse Transcriptases of Vpx Coding and Non-coding Lentiviruses*

**DOI:** 10.1074/jbc.M115.691576

**Published:** 2015-10-19

**Authors:** Gina M. Lenzi, Robert A. Domaoal, Dong-Hyun Kim, Raymond F. Schinazi, Baek Kim

**Affiliations:** From the ‡ Center for Drug Discovery, Department of Pediatrics, Emory University School of Medicine, Atlanta, Georgia 30322,; the § College of Pharmacy, Kyung-Hee University, Seoul 02447, South Korea,; the ¶ Veterans Affairs Medical Center, Decatur, Georgia 30033

**Keywords:** DNA replication, enzyme kinetics, lentivirus, macrophage, reverse transcription, SAMHD1, Vpx, dNTP

## Abstract

Among lentiviruses, HIV Type 2 (HIV-2) and many simian immunodeficiency virus (SIV) strains replicate rapidly in non-dividing macrophages, whereas HIV Type 1 (HIV-1) replication in this cell type is kinetically delayed. The efficient replication capability of HIV-2/SIV in non-dividing cells is induced by a unique, virally encoded accessory protein, Vpx, which proteasomally degrades the host antiviral restriction factor, SAM domain- and HD domain-containing protein 1 (SAMHD1). SAMHD1 is a dNTPase and kinetically suppresses the reverse transcription step of HIV-1 in macrophages by hydrolyzing and depleting cellular dNTPs. In contrast, Vpx, which is encoded by HIV-2/SIV, kinetically accelerates reverse transcription by counteracting SAMHD1 and then elevating cellular dNTP concentration in non-dividing cells. Here, we conducted the pre-steady-state kinetic analysis of reverse transcriptases (RTs) from two Vpx non-coding and two Vpx coding lentiviruses. At all three sites of the template tested, the two RTs of the Vpx non-coding viruses (HIV-1) displayed higher *k*_pol_ values than the RTs of the Vpx coding HIV-2/SIV, whereas there was no significant difference in the *K_d_* values of these two groups of RTs. When we employed viral RNA templates that induce RT pausing by their secondary structures, the HIV-1 RTs showed more efficient DNA synthesis through pause sites than the HIV-2/SIV RTs, particularly at low dNTP concentrations found in macrophages. This kinetic study suggests that RTs of the Vpx non-coding HIV-1 may have evolved to execute a faster *k*_pol_ step, which includes the conformational changes and incorporation chemistry, to counteract the limited dNTP concentration found in non-dividing cells and still promote efficient viral reverse transcription.

## Introduction

Lentiviruses such as human immunodeficiency virus Type 1 (HIV-1),^2^ HIV Type 2 (HIV-2), and simian immunodeficiency viruses (SIV) replicate in both activated and dividing CD4^+^ T cells and terminally differentiated/non-dividing myeloid cells such as macrophages and microglia, whereas other retroviruses such as gammaretroviruses (*i.e.* murine leukemia virus (MuLV) and feline leukemia virus), alpharetroviruses (*i.e.* avian myeloblastosis virus), and spumavirus (*i.e.* foamy virus) replicate only in dividing cells (1, 2). A key metabolic difference between dividing and non-dividing cells is the cellular deoxynucleotide triphosphate (dNTP) pool. Cellular dNTP biosynthesis is closely tied with the cell cycle; the expression of various enzymes involved in dNTP biosynthesis is specifically activated at G_1_/S and S phases to support chromosomal DNA replication, which consumes cellular dNTPs (3, 4). It is well established that cancer cells have higher dNTP concentrations than normal dividing cells due to cell cycle dysregulation (5, 6). Also, it was postulated that non-dividing cells including macrophages have lower dNTP concentrations than dividing cells due to lack of cell cycling and chromosomal DNA replication. However, the actual dNTP concentration of human primary macrophages was not available due to sensitivity limitations of available dNTP assays until we developed a highly sensitive method to determine the dNTP concentration in human primary macrophages (7). Indeed, we reported that human primary monocyte-derived macrophages have 50–200 times lower dNTP concentrations (20–40 nm) than activated CD4^+^ T cells (2–4 μm) (7, 8). Importantly, although HIV-1 replication and viral production are robust in activated CD4^+^ T cells, its replication in non-dividing macrophages is kinetically delayed (9, 10). Our studies demonstrate that the extremely low dNTP level found in macrophages mechanistically contributes to the delayed replication kinetics of HIV-1 in macrophages and non-dividing cells.

Unlike HIV-1, HIV-2 and many SIV strains replicate rapidly even in macrophages, and this efficient replication capability of HIV-2/SIV in macrophages is engineered by a virally encoded accessory protein, called viral protein X (Vpx) (11, 12). Two groups independently reported that Vpx induces the fast replication kinetics in non-dividing macrophages by proteasomally degrading a host myeloid specific anti-viral factor, SAM domain- and HD domain-containing protein 1 (SAMHD1) (13, 14). Later, SAMHD1 was reported to be a dNTPase that hydrolyzes dNTPs to deoxynucleosides and triphosphates (15), and indeed, our study revealed that SAMHD1 restricts reverse transcription during HIV-1 replication in macrophages by depleting cellular dNTPs and that the Vpx-mediated SAMHD1 degradation enhances reverse transcription by elevating cellular dNTPs in non-dividing macrophages (16). This Vpx-mediated dNTP elevation also facilitates viral replication in other non-dividing cell types including dendritic cells (17) and resting CD4^+^ T cells (18). Basically, Vpx coding HIV-2/SIV replicate in an abundant dNTP condition even in non-dividing cells by counteracting SAMHD1, whereas Vpx non-coding lentiviruses (HIV-1) always replicate under limited dNTP availability in non-dividing cells. This difference contributes to the delayed replication kinetics exhibited by HIV-1 in macrophages and other non-dividing target cells.

We previously reported that HIV-1 RT very efficiently synthesizes DNA especially at low dNTP concentrations, as compared with MuLV RT. Furthermore, the pre-steady-state kinetic data demonstrated that HIV-1 RT has a tighter dNTP binding affinity (*K_d_*) than MuLV RT (1). We suggested that the tight dNTP binding affinity of HIV-1 RT promotes synthesis of its proviral DNA in macrophages, which have a low dNTP concentration. Conversely, MuLV RT may not require tight dNTP binding nor DNA synthesis at low dNTP concentrations because MuLV does not infect non-dividing cells such as macrophages. This study suggests that the enzyme kinetics of the RT contribute to the cell tropism (dividing *versus* non-dividing cells) of retroviruses. This idea was further supported by our finding that RT of a SIV clone that preferentially replicates in activated CD4^+^ T cells where dNTP concentrations are high showed a reduced dNTP binding affinity, which results from a mutation (V148I), as compared with a parental virus that preferentially infects macrophages (19, 20).

Based on the findings that SAMHD1 mediates the dNTP depletion of macrophages, we reasoned that Vpx coding HIV-2/SIV replicate under increased dNTP conditions even in non-dividing cells by counteracting SAMHD1. In contrast, Vpx non-coding lentiviruses (HIV-1) must replicate under limited dNTP availability in non-dividing cells, which contributes to the delayed HIV-1 replication kinetics in the non-dividing viral target cell types. Indeed, our study on the dNTP utilization efficiency of 7 different HIV-1 RTs (Vpx non-coding) and 11 different HIV-2/SIV (Vpx coding) RTs revealed that the Vpx non-coding viral RTs tested showed more efficient DNA synthesis at low dNTP concentrations, as compared with the Vpx coding HIV-2/SIV RTs (21), which supports the idea that Vpx and SAMHD1 can influence RT enzyme kinetics. Here we investigated the mechanistic differences between these two groups of RT enzymes using pre-steady-state kinetic analysis, which can separately determine the dNTP binding affinity (*K_d_*) and the following conformational change/incorporation chemistry step (*k*_pol_ step). We observed that HIV-1 RT enzymes have a faster *k*_pol_ step, but similar dNTP binding affinity, as compared with the RTs tested from Vpx coding HIV-2/SIV.

## Experimental Procedures

### 

#### 

##### RT Expression and Purification

The HIV-1 Ug, HIV-1 Cy, HIV-2 Rod, and African green monkey SIV (SIVagm) 9063-2 RT clones were generously provided by the National Institutes of Health AIDS Reagent Program and V. Hirsch (NIAID). The RT sequences were previously cloned into pET28a (Novagen), and the N-terminal hexahistidine-tagged-p66/p66 homodimer RTs were subsequently expressed in *Escherichia coli* BL21 (DE3) pLysS (Stratagene) and purified as described previously (22) with the following changes. Cleared lysate was applied to nickel-nitrilotriacetic acid His·Bind Superflow resin (Millipore) equilibrated with a binding buffer containing 40 mm Tris-HCl, pH 7.5, 250 mm KCl, 20 mm imidazole, 5 mm MgCl_2_, 10% glycerol, and 5 mm β-mercaptoethanol. The column was washed for 20 column volumes with increased KCl (1 m final), and finally, proteins were eluted with increased imidazole (300 mm). Fractions containing His_6_-p66 were combined and then further purified on an HiPrep 16/60 Sephacryl S-200 HR (GE Healthcare) with a buffer containing 50 mm Tris-HCl, pH 7.5, 150 mm KCl, 20% glycerol, 0.25 mm EDTA, and 1 mm β-mercaptoethanol. The purity of the RT enzymes was typically greater than 95% as determined from SDS-PAGE. RT enzymes were flash-frozen in liquid nitrogen and stored at −80 °C until use.

##### Multiple dNTP Incorporation Assay

The primer extension assay was modified from a previously described assay (7). Briefly, a template/primer (T/P) was prepared by annealing a 5′ ^32^P-labeled 17-mer DNA primer (5′-CGCGCCGAATTCCCGCT-3′, Integrated DNA Technologies) to a 2.5-fold excess of 40-mer template RNA (5′-AAGCUUGGCUGCAGAAUAUUGCUAGCGGGAAUUCGGCGCG-3′, Integrated DNA Technologies). Assay mixtures (20 μl) contained 10 nm T/P, RT, and dNTP at the concentrations specified in each figure legend. Reaction mixtures were incubated at 37 °C for 5 min and then terminated for analysis. This reaction condition allows multiple rounds of primer extension, and all measured enzyme activity was normalized for 50% extension at the highest dNTP concentration. Products were resolved using 14% polyacrylamide/8 m urea gels and visualized using a PharosFX (Bio-Rad).

##### Pre-steady-state Burst Experiments

Pre-steady-state burst experiments were performed using an RQF-3 rapid quench-flow apparatus (KinTek Corp.) to determine the active concentration of the purified RT enzymes. The T/P was prepared as above and consisted of a 48-mer DNA template (5′-CGAGCTAAGCGCTTGACCGCAGAACATTGCTAGCGGGAATTCGGCGCG-3′) and a 21-mer primer (5′-CGCGCCGAATTCCCGCTAGCA-3′, template:primer ratio of 2.5:1). In this experiment, 300 μm dATP and 10 mm MgCl_2_ were rapidly mixed with RT (100 nm total protein) prebound to T/P (300 nm). All concentrations represent the final concentrations after mixing. The reactions were quenched at various time points with 0.3 m (final) EDTA. Products were then separated on a 20% polyacrylamide/8 m urea gel, visualized using a PharosFX (Bio-Rad), and quantified with Molecular Imager FX software (Bio-Rad). Product formation was fit to the burst equation

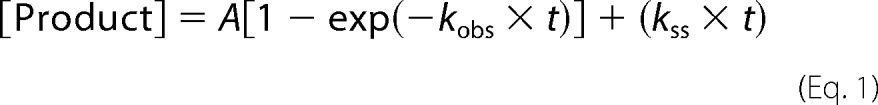
 in which *A* is the amplitude of the burst, *k*_obs_ is the observed first-order burst rate constant, and *k*_ss_ is the linear steady-state rate constant (23).

##### Single-turnover Experiments

Rapid chemical quench experiments were performed as described previously with an RQF-3 rapid quench-flow apparatus (KinTek Corp.) to examine the transient kinetics associated with incorporating a single nucleotide onto three different T/Ps (23, 24). All reactions used the same 40-mer RNA template, but each annealed with different ^32^P-labeled DNA primer. Site 1 (no pause) used the 17-mer (5′-CGCGCCGAATTCCCGCT-3′, Integrated DNA Technologies), Site 2 (unique pause) used a 22-mer (5′-CGCGCCGAATTCCCGCTAGCAA-3′), and Site 3 (conserved pause) used a 30-mer (5′-CGCGCCGAATTCCCGCTAGCAATATTCTGC-3′). The reactions were carried out by rapid mixing of a solution containing the preincubated complex of 250 nm of the RT (active concentration) and 50 nm T/P with a solution of 10 mm MgCl_2_ and varying concentrations of dNTP in the presence of 50 mm Tris-HCl, pH 7.8, and 50 mm NaCl at 37 °C. Reactions were quenched, separated, visualized, and quantified as described above. Product formation was fit to a non-linear regression curve equation

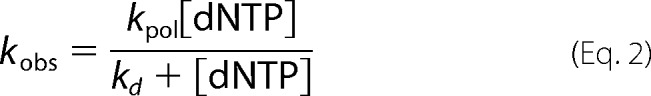
 where *k*_obs_ is the observed pre-steady-state burst rate, *k*_pol_ is the maximum rate of incorporation, and *K_d_* is the equilibrium dissociation constant for the dNTP (23, 25, 26).

##### Generation of Viral RNA Templates

RNA templates previously used for strand transfer studies were generated as described previously (27, 28). Briefly, TAR and part of the *pol* gene were first PCR-amplified using D3 as a template. The PCR product was then agarose gel-purified using a Wizard SV Gel Clean-Up Kit (Promega). Finally, both RNAs were *in vitro* transcribed with these PCR products as DNA templates using the MEGAshortscript kit (Ambion). *In vitro* transcribed products were then gel-purified by 10% polyacrylamide/8 m urea PAGE and UV shadowing.

##### Multiple dNTP Incorporation on Viral Template

The primer extension assays were performed similarly as described above, but using *in vitro* transcribed viral RNA (TAR and shortened *pol* gene) as RNA templates. ^32^P-labeled 20-mer DNA primers (5′-ACAGACGGGCACACACTACT-3′ and 5′-GACGCATGTGACTGATATCC-3′ for TAR and Pol RNA templates, respectively) were annealed onto these RNA templates to form each T/P pair. Assays were carried out as described above except enzyme activity was normalized by using the same active concentration (200 nm).

## Results

### 

#### 

##### dNTP Concentration-dependent DNA Synthesis Efficiency of RTs from Vpx Coding and Non-coding Lentiviruses

**FIGURE 1.**
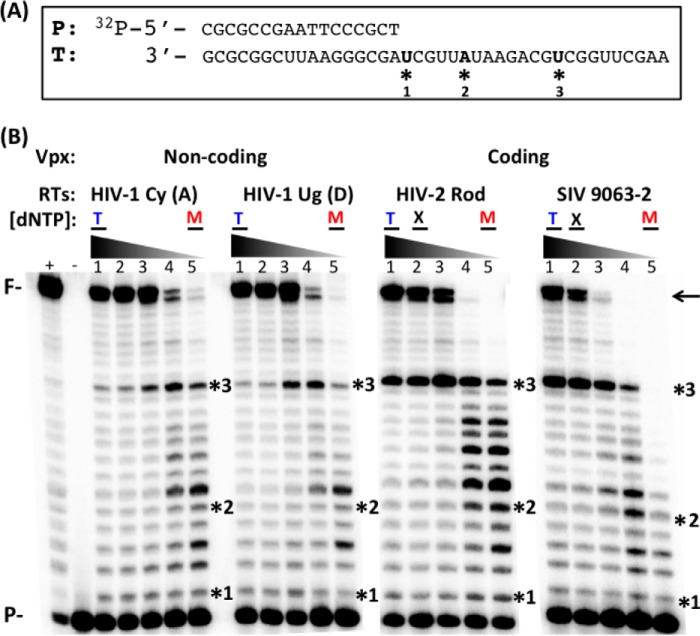
**dNTP concentration-dependent DNA synthesis of RT enzymes from Vpx coding and non-coding lentiviruses.** *A*, template (*T*) and primer (*P*) used in this study. 5′ ^32^P-labeled 17-mer DNA primer was annealed to 40-mer RNA template. The three sites (*) used for pre-steady-state analysis are indicated. *B*, the T/P was extended by four purified RT enzymes from either Vpx non-coding or Vpx coding lentiviruses under the conditions described under “Experimental Procedures” at different dNTP concentrations (*lanes 1–5*: 1 μm, 500 nm, 200 nm, 100, nm, 50 nm). The RTs used from Vpx non-coding viruses were HIV-1 Cy (subtype A) and HIV-1 Ug (subtype D), and the RTs used from Vpx coding viruses were HIV-2 Rod and SIV 9063-2. RT activity used in this assay generated ∼50% primer extension at the high dNTP concentration found in activated CD4^+^ T cells (T and *lane 1*) as determined by the quantitation of the 40-bp fully extended product (*F* and ←). The three sites analyzed for the pre-steady-state kinetic study are also marked with *. (+): 50 μm dNTP positive control; (−): no dNTP control. T: dNTP concentration found in activated CD4^+^ T cells; M: dNTP concentration found in macrophages; X: dNTP concentration found in macrophages treated with Vpx (16). *F*: fully extended products; *P*: primer and unextended substrate.

Because Vpx non-coding and coding lentiviruses are both able to replicate in macrophages with significant dNTP availability differences, we tested whether RTs from Vpx coding and non-coding lentiviruses display different concentration-dependent activity profiles. To test this, we cloned, overexpressed, and purified homodimeric p66 RTs from two HIV-1 strains of different subtypes (A and D: Vpx non-coding), HIV-2 Rod, and SIVagm 9063-2 (Vpx coding). First we examined the effect of dNTP concentration on the RNA-dependent DNA polymerization activity of these purified RT proteins using a 40-mer RNA template (*T*) annealed to a 5′ ^32^P-labeled 17-mer DNA primer (*P*, Fig. 1*A*) and varying dNTP concentrations observed in activated/dividing CD4^+^ T cells (1 μm, T in Fig. 1*B*), non-dividing macrophages (50 nm, M), and non-dividing macrophages with Vpx (500 nm, X). We initially determined the amount of the RT enzymes showing ∼50% primer extension at 1 μm dNTPs (T: T cell concentration) as calculated by the ratio of unextended primer (*P*) to fully extended primer (*F*) in a 5-min incubation at 37 °C. Then, the same reactions were repeated with decreasing concentrations of dNTPs down to 50 nm (M). As shown (Fig. 1*B*), the two HIV-1 RTs were able to generate the full-length product (see *arrow*), even at low dNTP concentrations found in macrophages, and this is consistent with our previous observations for other HIV-1 RT variants (21). The HIV-2 and SIV RTs also efficiently fully extended the primer in both T cell dNTP concentration and the dNTP concentration found in macrophages treated with Vpx (X, 500 nm (16)). However, the HIV-2 and SIV RTs tested generated 5–10 times less fully extended product at low dNTP concentrations found in macrophages (M) as compared with HIV-1 RTs. Pausing (see Sites 2 and 3, * in Fig. 1, *A* and *B*) is generated by the kinetic delay of dNTP incorporation and is more significant in the HIV-2 and SIV RT proteins as compared with the HIV-1 RT enzymes (unpaired Student's *t* test between RTs from Vpx non-coding and coding viruses, *p* < 0.05). This initial analysis shown in Fig. 1 suggests that RTs from these two groups of lentiviruses (Vpx non-coding and coding) have different DNA polymerase activity profiles, especially at low dNTP concentrations found in non-dividing macrophages.

##### Pre-steady-state Kinetic Analysis of the Four RT Enzymes at Three Different Sites

**FIGURE 2.**
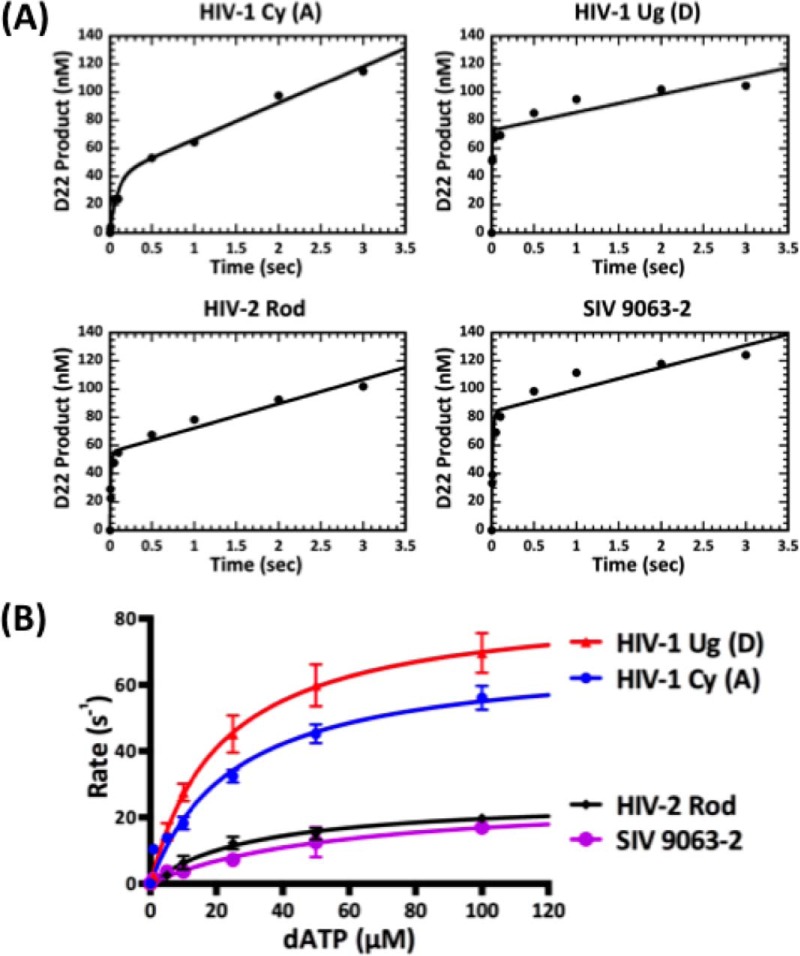
**Active concentration determination and the pre-steady-state dATP incorporation kinetics of RT enzymes from Vpx non-coding and coding lentiviruses at Site 1 of the 40-mer RNA template.** *A*, pre-steady-state burst kinetics of incorporation of dATP onto the T/P described under “Experimental Procedures” by the four RT enzymes. The *solid line* represents a fit to a burst equation. Burst experiments were repeated 2–3 times for each enzyme, and a representative curve for each enzyme is shown. Percentages of the active concentrations for HIV-1 Cy, HIV-1 Ug, HIV-2 Rod, and SIV 9063-2 are 40, 73, 55, and 84%, respectively. *B*, pre-steady-state incorporation rates of the four RT proteins at varying dATP concentration (1–100 μm) at Site 1 of the T/P described in the legend for Fig. 1 were plotted. The fit to the data gave the following *K_d_* (dNTP binding constant) and *k*_pol_ (maximum incorporation rate) values, respectively: HIV-1 Cy, 30.9 μm and 67.2 s^−1^; HIV-1 Ug, 28.4 μm and 86.6 s^−1^; HIV-2 Rod, 30.4 μm and 29.6 s^−1^; and SIV 9063-2, 40.0 μm and 22.3 s^−1^ (see Table 1 for detail). Experiments were repeated 3–7 times for the four enzymes at Site 1, and the average is shown with *error bars* representing S.E.

To understand the mechanistic discrepancy in the DNA synthesis kinetics between the RTs from Vpx non-coding and coding lentiviruses, we sought to determine the dNTP binding affinity (*K_d_*) and incorporation rate (*k*_pol_) for each RT enzyme. To determine the active enzyme concentration of the four RT proteins, we first used pre-steady-state burst experiments (molar excess of T/P) with the T/P that does not induce RT pausing (see “Experimental Procedures”). We observed typical burst kinetics for all four RTs (Fig. 2*A*) followed by the slow steady-state rate, giving a ratio of active protein ranging from 50 to 75%. This indicates that the mechanistic pathway was not changed for the enzymes tested and that a slow step following the chemistry is limiting the overall reaction pathway.

Using all four enzymes normalized for active concentration, we employed single turnover experiments to determine each enzyme's binding affinity (*K_d_*) and incorporation rate (*k*_pol_) at Site 1 of the T/P used in Fig. 1. We used concentrations of dNTPs ranging from 1 to 100 μm and determined the rate of single nucleotide incorporation at each concentration. Those rates were plotted against the dNTP concentration to determine both the maximum rate of incorporation and dNTP binding affinity. Fig. 2*B* displays the binding curves for Site 1 for the four enzymes. As evident from the graph, the Vpx non-coding HIV-1 RTs plateau at a higher rate of incorporation as compared with the Vpx coding HIV-2 Rod and SIV 9063-2. However the dNTP binding affinities for all four enzymes at Site 1 are not statistically different (Table 1). These data from Site 1 suggest that the RTs from HIV-1 may have higher rates of dNTP incorporation but similar dNTP binding affinities to RTs from Vpx coding SIV and HIV-2 lentiviruses.

**TABLE 1:** ***k*_pol_, *K_d_*, *k*_pol_/*K_d_* values of Vpx non-coding and Vpx coding RTs at three different sites on the 40-mer RNA template** -Fold changes between two groups of the RT enzymes are indicated in parentheses. Significant differences in -fold changes are bolded. Statistical significance from an unpaired Student's *t* test is indicated as: NS, not significant; *, *p* < 0.05; **, *p* < 0.01; ***, *p* < 0.001.

Site	RTs	*k*_pol_	*K_d_*	*k*_pol_/*K_d_*
		*s*^−*1*^	μ*m*	*s*^−*1*^ μ*m*^−*1*^
*1 (No pause)	Vpx non-coding (HIV-1 Cy, HIV-1 Ug)	79 ± 15 (3.0×)*	29 ± 5.4 (0.7×) NS	2.7 ± 0.34 (3.6×)**
	Vpx coding (HIV-2 Rod, SIV 9063–2)	27 ± 3.2	39 ± 8.4	0.76 ± 0.15
*2 (Unique pause)	Vpx non-coding (HIV-1 Cy, HIV-1 Ug)	290 ± 31 (4.1×)***	71 ± 11 (1.4×) NS	4.1 ± 0.26 (3.2×)***
	Vpx coding (HIV-2 Rod, SIV 9063–2)	69 ± 17	51 ± 7.8	1.3 ± 0.19
*3 (Conserved pause)	Vpx non-coding (HIV-1 Cy, HIV-1 Ug)	43 ± 4.5 (2.0×)*	22 ± 4.6 (0.4×) NS	2.4 ± 0.52 (4.8×)*
	Vpx coding (HIV-2 Rod, SIV 9063–2)	22 ± 5.1	50 ± 15	0.49 ± 0.10

**FIGURE 3.**
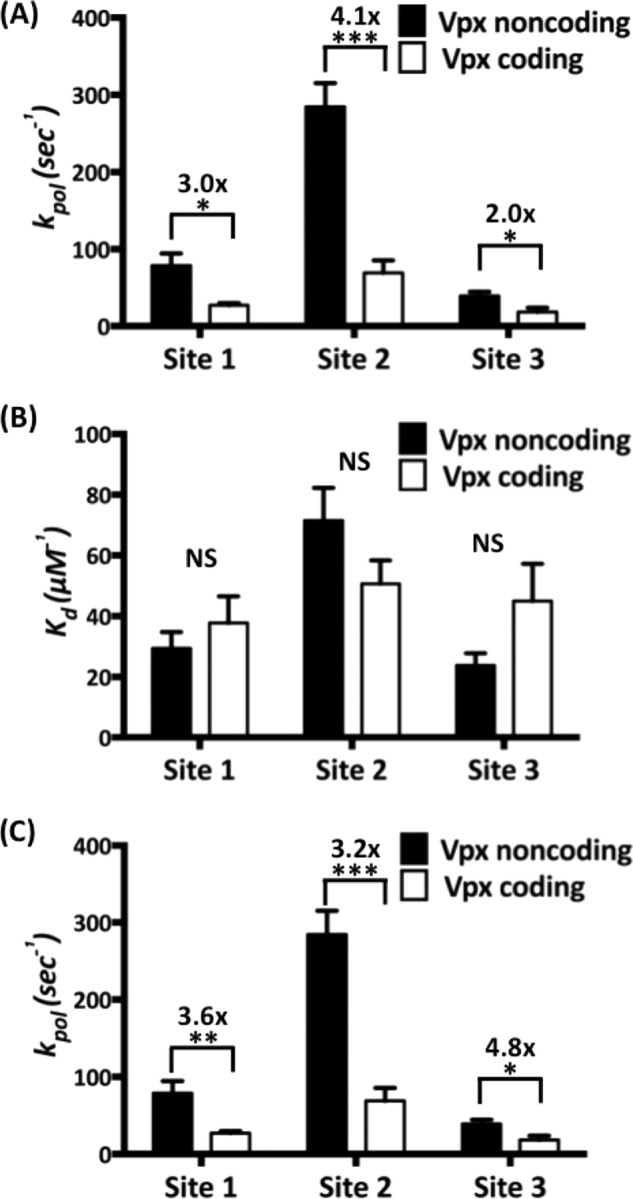
***k*_pol_, *K_d_*, and *k*_pol_*/K_d_* comparison of the four RT enzymes at three different sites on the 40-mer RNA template.** *A–C*, the maximum incorporation rates (*k*_pol_) (*A*), the dNTP binding affinity (*K_d_*) (*B*), and the incorporation efficiency (*k*_pol_*/K_d_*) (*C*) of RT protein from Vpx non-coding (*black bars*) and Vpx coding (*open bars*) lentiviruses at the three different sites described in the legend for Fig. 1 were determined 3–7 times, and the average values are shown with *error bars* representing S.E. -Fold changes are indicated by *brackets* above the bars, and statistical significance from an unpaired Student's *t* test is indicated as: *NS*, not significant; *, *p* < 0.05; **, *p* < 0.01; ***, *p* < 0.001.

Next, we repeated the experiments at two pause sites along the same template (Sites 2 and 3, Fig. 1). Site 2 is a unique pause site where only HIV-2/SIV RTs showed pausing, whereas Site 3 is a common pause site where all four RTs experienced kinetic delays (Fig. 1*B*). All *K_d_* and *k*_pol_ values of the four RTs at the three different sites are shown in Table 1. When these values were compared (Fig. 3), the maximum rate of incorporation (*k*_pol_, Fig. 3*A*) was 2–4-fold higher for RTs from Vpx non-coding lentiviruses as compared with RTs from Vpx coding lentiviruses (Fig. 3*A*). Indeed, there are no significant differences in dNTP binding affinity for the two classes of RTs at any of the three sites tested (Fig. 3*B*). The overall dNTP incorporation efficiency, which is a ratio of incorporation rate to dNTP binding affinity (*k*_pol_/*K_d_*), is also 3–5-fold higher for the HIV-1 RTs as compared with HIV-2/SIV RTs at all three sites of the T/P tested (Fig. 3*C*). Overall, these results suggest that the *k*_pol_ step, which includes two sequential sub-steps, 1) conformational change and 2) dNTP incorporation chemistry (29), is significantly faster in RTs from Vpx non-coding lentiviruses as compared with Vpx coding lentiviruses. Note that the conformational change step, which occurs after the dNTP binding and before incorporation chemistry, is a rate-limiting step for many DNA polymerases (29, 30).

##### dNTP Concentration-dependent DNA Synthesis with Viral RNA Templates Harboring RNA Structure-induced RT Pause Sites

**FIGURE 4.**
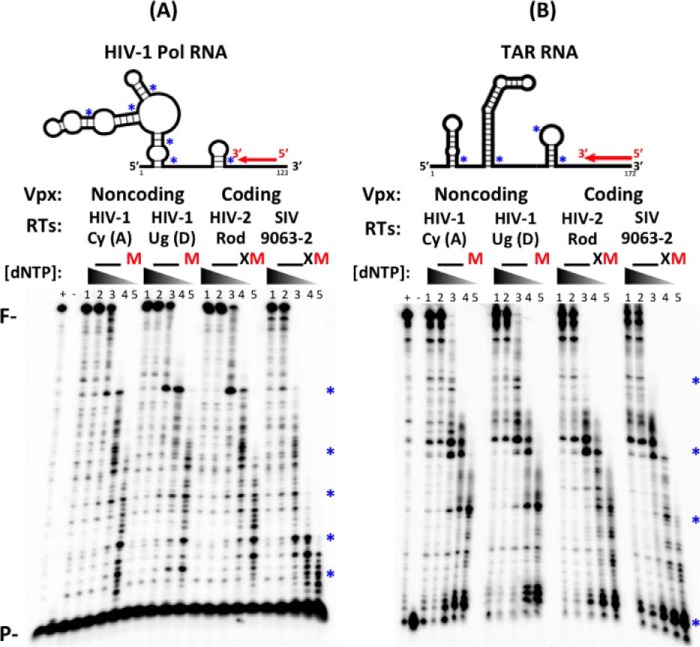
**dNTP concentration-dependent RNA-dependent DNA synthesis of the four RT enzymes with two long viral RNA templates.** *A* and *B*, schematic showing 5′ ^32^P-labeled 20-mer primer (*P*) annealed to HIV-1 Pol (*A*, *top*) or TAR (*B*, top) RNA template. Template structure was based on Mfold prediction for lowest free energy (36). The predicted bottoms of each stem-loop structure in these RNA templates were marked by *. The 5′ ^32^P-labeled primers annealed to the Pol or TAR RNAs (*A* and *B*) were extended by an equal active concentration of the four purified RT proteins at five different dNTP concentrations (*lanes 1–5*: 50, 10, 1, 0.25, 0.1 μm), which are close to the dNTP concentrations found in activated T cells (__), macrophages (M), and macrophages treated with Vpx (X), respectively. * indicates pause sites produced by kinetic delays of dNTP incorporations at lower dNTP concentrations near the bottom of each stem-loop structure predicted in the RNA templates. (+): 100 μm dNTP positive control; (−): no dNTP control. *F*: fully extended products; *P*: primer and unextended substrate.

RT-mediated RNA-dependent DNA synthesis kinetics are also affected by the secondary structure of RNA templates (31). Basically, RT pauses at the bottom of the stem-loop structures found in RNA templates, leading to kinetic delays, particularly at short time points (28). This RT pausing is known to trigger RT strand transfer and recombination after the degradation of the RNA template by the RNase H activity of RT (32, 33). To assess whether the two groups of lentiviral RTs also display different dNTP concentration-dependent DNA synthesis efficiencies at the RNA structure-induced pause sites, we performed the primer extension assay using long RNA templates that harbor strong secondary structures and induce RT pausing. First, we chose a sequence of the *pol* gene that is known to have multi-branched loops (27, 34) and the highly conserved 5′-UTR TAR stem-loop (35) as illustrated in the *top panels* of Fig. 4, *A* and *B* (36). When primers annealed to these long RNA templates were extended with the same amount of active RT enzyme (Fig. 4, *A* and *B*, *bottom panels*), we observed pause products at several key sites (see * in Fig. 4) at or near the bottom of the stem-loop structures predicted in both RNA templates (Fig. 4, *A* and *B*), even at some high dNTP concentrations found in T cells (1 and 10 μm, *lanes 2* and *3*, respectively; __ in Fig. 4*B*). This RT pausing became more evident for all four RT enzymes at low dNTP concentrations found in macrophages (M) and macrophages treated with Vpx (X). However, it is clear that RTs from Vpx coding lentiviruses generated more incomplete short products at the pause sites, particularly at low dNTP concentrations (see X and M), as compared with RTs from non-coding lentiviruses (unpaired Student's *t* test between RTs from Vpx non-coding and coding viruses at macrophage conditions for both Pol and TAR templates, *p* < 0.05). These results indicate that RTs from Vpx coding SIV and HIV-2 experience more pausing and kinetic delay due to decreased dNTP concentrations than RTs from HIV-1 during reverse transcription of structured viral RNA templates.

## Discussion

Cellular replicative DNA polymerases always operate at high dNTP concentrations found in dividing cells because they duplicate chromosomal DNA only during S phase of the cell cycle where dNTP biosynthesis is activated. Importantly, the steady-state *K_m_* values of many cellular replicative DNA polymerases are close to or above the cellular dNTP concentrations found in the dividing cells (37, 38), supporting the idea that there is evolutionary crosstalk between the enzyme kinetics of the cellular DNA polymerases and cellular dNTP concentrations.

In 2004, we reported that human primary macrophages harbor extremely low dNTP concentrations (20–40 nm), as compared with activated CD4^+^ T cells (1–10 μm) (7), and in 2012, we reported that host SAMHD1 protein, which is a dNTPase, is responsible for the low dNTP concentrations found in macrophages (16). Therefore, RTs of lentiviruses encounter two vastly different cellular dNTP environments in dividing (activated CD4^+^ T cells) *versus* non-dividing viral target cell types (macrophages, dendritic cells, and resting CD4^+^ T cells). Indeed, a series of our biochemical and virological studies suggested that lentiviral RTs may have evolved to efficiently synthesize DNA even at low dNTP concentrations to support viral reverse transcription in non-dividing viral target cells (21, 39, 40). This was supported by the biochemical finding that RTs of gammaretroviruses or alphaviruses such as MuLV, feline leukemia virus, and avian myeloblastosis virus, which replicate only in dividing cells, synthesize DNA efficiently only at the high dNTP concentrations found in dividing cells (1, 2). Our pre-steady-state kinetic study revealed that HIV-1 RT has a higher dNTP binding affinity than MuLV RT (1), supporting that lentiviral RTs may have evolved to bind dNTP tightly to support efficient reverse transcription at the low cellular dNTP concentration found in non-dividing macrophages.

However, some Vpx coding lentiviruses replicate at higher cellular dNTP concentrations even in macrophages because Vpx elevates cellular dNTP concentration close to the dNTP concentration found in dividing cells (*i.e.* activated CD4^+^ T cells) by counteracting the host SAMHD1 protein (16). This led us to test whether Vpx coding and non-coding lentiviral RTs display different DNA synthesis efficiencies at low dNTP concentrations. Indeed, our previous steady-state kinetic analysis with RTs from 19 different lentiviruses revealed that the Vpx non-coding lentiviral RTs such as HIV-1 RTs have lower *K_m_* values than the Vpx coding lentiviral RTs (HIV-2 and SIV RTs) (21).

Our pre-steady-state kinetic data with four different RTs at multiple sites supports that the polymerases from these Vpx coding and non-coding lentiviruses display different *k*_pol_ values, rather than *K_d_* values. This finding was rather unexpected because the two SIV RT variants that we previously characterized (SIVmne CL8 and 170) showed different *K_d_* values with similar *k*_pol_ values (20), and the T-cell tropic SIVmne170 RT gained the V148I mutation near the active site that reduced its dNTP binding affinity (19). This study led us to hypothesize that SIVmne170 RT may have lost the tight dNTP binding affinity because this virus only infects activated CD4^+^ T cells where dNTP concentrations are high. Therefore, the pre-steady-state kinetic data from this study and previous studies support that both *K_d_* and *k*_pol_ steps can vary among lentiviral RTs, and these distinct mechanistic variations may contribute to the cell tropism of lentiviruses (dividing *versus* non-dividing cells).

The *K_d_* values, which represent the binding affinity to the incoming nucleotide, are the first reported for HIV-1 subtypes A and D, HIV-2, and SIV RTs. Comparing these values with previously reported pre-steady-state results, the binding affinities are slightly higher than those published for HIV-1 subtype B. Previous research has shown that using a homodimeric enzyme and an RNA template has a weaker binding affinity as compared with using a heterodimeric enzyme and a DNA template (23, 41). In addition to subtype differences, we hypothesize that the RNA template and p66 homodimers could contribute to the higher binding affinity.

Importantly, the *k*_pol_ values of DNA polymerases represent two sequential sub-steps following the dNTP binding to the active site (*K_d_* step), 1) conformational change and 2) catalysis. Also, it is well established that the conformational change step, which occurs after dNTP binding and before incorporation chemistry, is a rate-limiting step during the overall dNTP incorporation reaction (29, 30). Then, which of these two sub-steps (or both) varies between the tested polymerases from Vpx coding and non-coding viruses? To postulate on this question, we compared the sequences of the four RTs tested with HIV-1 HXB2 (subtype B) in the fingers and palm domains that contain many residues important for DNA polymerization including dNTP binding and chemistry of DNA synthesis (*i.e.* metal binding). All residues known to be involved in dNTP binding (Asp-113, Ala-114, Tyr-115, Gln-151, lys-65, Arg-72) and metal binding/catalysis (Asp-110, Tyr-183, Met-184, Asp-185, Asp-186) are conserved among these four RTs (42), indirectly supporting the similar *K_d_* values for these four enzymes. Val-148 is also involved in dNTP binding and highly conserved, but our previous research has shown that the Cys-148 of SIV 9063-2 has no effect on dNTP binding affinity (43). Although all key residues important for DNA synthesis are conserved among these four RTs, there are significant sequence variations throughout the enzymes (44–47), possibly implying that these sequence variations may affect the overall conformational change efficiency rather than the chemical catalysis step and may lead to efficiency differences in proviral DNA synthesis in the low cellular dNTP environments found in non-dividing viral target cell types. Future studies will elucidate whether conformational change and/or chemical catalysis differ between RTs from Vpx non-coding and coding lentiviruses.

## Author Contributions

G. M. L. purified the RTs, performed the experiments, analyzed the data, and edited the paper. R. A. D. helped with the pre-steady-state experiments and contributed training and materials for kinetic assays. R. F. S. and D. H. K. conceived the experiments. B. K. conceived and designed the experiments and wrote the paper. All authors read and approved the final manuscript.

## Footnotes

2 The abbreviations used are:

HIV-1: HIV Type 1
HIV-2: HIV Type 2
SIV: simian immunodeficiency virus(es)
RT: reverse transcriptase
dNTP: deoxynucleotide triphosphate
SAMHD1: SAM domain- and HD domain-containing protein 1
SAM domain: sterile α motif domain
HD: histidine-aspartic domain
SIVagm: African green monkey SIV
T/P: template/primer
Vpx: viral protein X
MuLV: murine leukemia virus
TAR: transactivation response element.
